# Knockdown of circDENND4C inhibits glycolysis, migration and invasion by up-regulating miR-200b/c in breast cancer under hypoxia

**DOI:** 10.1186/s13046-019-1398-2

**Published:** 2019-09-05

**Authors:** Shasha Ren, Jiuzhou Liu, Yun Feng, Zhenyu Li, Liang He, Leilei Li, Xiaozhong Cao, Zhenghua Wang, Yanwu Zhang

**Affiliations:** 1 Department of Thyroid and Breast Surgery, the Luoyang Central Hospital Affiliated to Zhengzhou University, No. 288 Zhongzhou Middle Road, Xigong District, Luoyang, China; 2 Department of Breast Surgery, the Third Affiliated Hospital of Zhengzhou University, No. 7 Kangfuqian Street, Erqi District, Zhengzhou, 450052 China

**Keywords:** Breast cancer, circDENND4C, miR-200b, miR-200c, Glycolysis, Migration

## Abstract

**Background:**

Hypoxia is a key feature of breast cancer, which affects cancer development, metastasis and metabolism. Previous studies suggested that circular RNAs (circRNAs) could participate in cancer progression and hypoxia regulation. This study aimed to investigate the role of circRNA differentially expressed in normal cells and neoplasia domain containing 4C (circDENND4C) in breast cancer progression under hypoxia.

**Methods:**

Forty-three patients with breast cancer were involved in this study. Breast cancer cell lines MDA-MB-453 and SK-BR-3 were cultured under hypoxia (1% O_2_) for experiments in vitro. The expression levels of circDENND4C, microRNA-200b (miR-200b) and miR-200c were measured by quantitative real-time polymerase chain reaction. Glycolysis was investigated by glucose consumption, lactate production and hexokinase II (HK2) protein level. Migration and invasion were evaluated via trans-well assay and protein levels of matrix metallopeptidase 9 (MMP9) and MMP2. The interaction between circDENND4C and miR-200b or miR-200c was explored by bioinformatics analysis, luciferase assay and RNA immunoprecipitation. Murine xenograft model was established to investigate the anti-cancer role of circDENND4C in vivo.

**Results:**

circDENND4C highly expressed in breast cancer was up-regulated in response to hypoxia. Knockdown of circDENND4C decreased glycolysis, migration and invasion in breast cancer cells under hypoxia. circDENND4C was validated as a sponge of miR-200b and miR-200c. Deficiency of miR-200b or miR-200c reversed the suppressive effect of circDENND4C knockdown on breast cancer progression. Moreover, silence of circDENND4C reduced xenograft tumor growth by increasing miR-200b and miR-200c.

**Conclusion:**

circDENND4C silence suppresses glycolysis, migration and invasion in breast cancer cells under hypoxia by increasing miR-200b and miR-200c.

**Electronic supplementary material:**

The online version of this article (10.1186/s13046-019-1398-2) contains supplementary material, which is available to authorized users.

## Background

Breast cancer is the most common malignancy with leading cause of death from cancer in women worldwide [1]. Hypoxia is an important feature of solid tumors, which contributes to progression of breast cancer and increases the risk of metastasis and mortality [2]. Under condition of hypoxia, glycolysis could maintain survival of cancer cells and promote cell progresses, such as proliferation, migration and invasion [3]. Hence, it is urgent to explore new mechanism underlying breast cancer progression under hypoxia.

Circular RNAs (circRNAs) are a class of single-stranded noncoding RNA molecules, which play essential roles in development and diagnosis of human diseases and cancers [4, 5]. Moreover, circRNAs have been regarded as potential therapeutic targets in breast cancer [6]. Xu et al. reported that circRNA transcriptional adaptor 2A (circTADA2A) inhibits cell proliferation, migration and invasion by regulating microRNA-203a-3p/suppressor of cytokine signaling 3 (SOCS3) axis in breast cancer [7]. Furthermore, Zhang et al. suggested that circRNA has_circ_0052112, generated from zinc finger 83, could promote cell migration and invasion by sponging microRNA-125a-5p in breast cancer [8]. CircRNA differentially expressed in normal cells and neoplasia domain containing 4C (circDENND4C) is a hypoxia-associated RNA molecule [9]. Notably, previous study indicated that high expression of circDENND4C promotes cell proliferation in breast cancer under hypoxia condition [10]. However, the role and mechanism of circDENND4C in breast cancer progression remain largely unknown.

CircRNAs usually exhibit their biological roles by acting as microRNA (miRNA) inhibitors or sponges by binding the seed sites to quench the normal function of miRNA in human cancers [11]. miRNAs are a class of small noncoding RNAs with 18–25 nucleotides, which play as promising targets for diagnosis, prognosis and therapeutics of breast cancer [12]. Previous works have demonstrated that miR-200b and miR-200c could serve as important tumor suppressor by inhibiting cell proliferation, migration and invasion in breast cancer [13–17]. More importantly, the complementary sites between circDENND4C and miR-200b or miR-200c predicted by bioinformatics analysis using starBase stimulated us to hypothesize that miR-200b and miR-200c might be required for circDENND4C-mediated progression of breast cancer.

In the current study, we measured the expression level of circDENND4C and investigated its biological role in glycolysis, migration and invasion in breast cancer cells under hypoxia. Moreover, we explored whether the regulatory mechanism was associated with miR-200b and miR-200c.

## Materials and methods

### Patients and tissues

A total of 43 patients with breast cancer were recruited from the Third Affiliated Hospital of Zhengzhou University. All participants have provided the written informed consents. The clinical features of patients were summarized in Table 1. The paired tumor tissues and corresponding adjacent normal samples were obtained prior to any other treatment and stored at -80 °C until used. The protocol of this study was approved by the ethics committee of the Third Affiliated Hospital of Zhengzhou University.

**Table 1:** Association between circDENND4C and the clinicopathological characteristics of breast cancer

	*N* (%)	circDENND4C level		*P* value
		High (%)	Low (%)	
Age(years)				*P* > 0.05
≥ 55	24 (55.8)	13 (54.2)	11 (45.8)	
< 55	19 (44.2)	10 (52.6)	9 (47.4)	
Menopause				*P* > 0.05
No	26 (60.5)	14 (53.8)	12 (46.2)	
Yes	17 (39.5)	9 (52.9)	8 (47.1)	
TNM stage				*P* < 0.05
I-II	23 (53.5)	8 (34.8)	15 (65.2)	
III-IV	20 (46.5)	15 (75.0)	5 (25.0)	
Lymph node metastasis				*P* < 0.05
No	22 (51.2)	8 (36.4)	14 (63.6)	
Yes	21 (48.8)	15 (71.4)	6 (28.6)	
Tumor size				*P* < 0.05
≥ 2 cm	25 (58.1)	17 (68.0)	8 (32.0)	
< 2 cm	18 (41.9)	6 (33.3)	12 (66.7)	
Subtype				*P* > 0.05
TNBC	19 (44.2)	10 (52.6)	9 (47.4)	
HER2	5 (11.6)	3 (60.0)	2 (40.0)	
Luminal A	8 (18.6)	4 (50)	4 (50)	
Luminal B	11 (25.6)	6 (54.5)	5 (45.5)	

Abbreviations: *TNM* tumor node metastasis, *TNBC* triple negative breast cancer, *HER2* human epidermal growth factor receptor 2

### Cell culture, hypoxia stimulation and cell transfection

Breast cancer cell lines (MDA-MB-453 and SK-BR-3) and normal human breast epithelial cells MCF-10A were purchased from the BeNa Culture Collection (Beijing, China), and cultured in RPMI-1640 medium (Gibco, Carlsbad, CA, USA) plus 10% fetal bovine serum at 37 °C with 5% CO_2_. For hypoxia stimulation, MDA-MB-453 and SK-BR-3 cells were growth in a hypoxia chamber with 1% O_2_ for various exposure times (0, 3, 6, 12, 24 and 48 h).

Small interfering RNA (siRNA) against circDENND4C (si-circ) (5′-AAGUAGCACUGCUCUUCAAAA-3′), siRNA negative control (si-NC) (5′-UCUCCGAACGUGUCACGUTT-3′), pcDNA-based circDENND4C overexpression vector (circ), pcDNA vector, miR-200b mimic (miR-200b) (5′-UAAUACUGCCUGGUAAUGAUGA-3′), miR-200c mimic (miR-200c) (5′-UAAUACUGCCGGGUAAUGAUGGA-3′), mimic negative control (miR-NC) (5′-UUCUCCGAACGUGUCACGUTT-3′), miR-200b inhibitor (anti-miR-200b) (5′-UCAUCAUUACCAGGCAGUAUUA-3′), miR-200c inhibitor (anti-miR-200c) (5′-UCCAUCAUUACCCGGCAGUAUUA-3′) and inhibitor negative control (anti-miR-NC) (5′-UUCUCCGAACGUGUCACGUTT-3′) were generated from Genepharma (Shanghai, China). When reaching 60% confluence, MDA-MB-453 and SK-BR-3 cells were transfected with 30 nM oligonucleotides or 200 ng vector using Lipofectamine 3000 (Invitrogen, Carlsbad, CA, USA) following the manufactures’ instructions. After transfection for 24 h, MDA-MB-453 and SK-BR-3 cells were collected for subsequent analyses.

### Quantitative real-time polymerase chain reaction (qRT-PCR)

Trizol reagent (Thermo Fisher Scientific, Waltham, MA USA) was used for RNA isolation from tissues or cells following the manufacturer’s protocols. For detecting level of circDENND4C, total RNA was treated by RNase R (Geneseed, Guangzhou, China) to improve purity of circRNA. The miScript Reverse Transcription Kit (Qiagen, Dusseldorf, Germany) was used for reverse transcription with 500 ng total RNA treated by RNase R or not and qRT-PCR was performed with the diluted cDNA products, special primers and SYBR Green mix (Thermo Fisher Scientific). The amplification conditions were: 95 °C for 1 min, 40 cycles of 95 °C for 10 s and 60 °C for 30 s. The primers were as follows: circDENND4C (Forward, 5′-GGGGCAGCAGTATTGTGAAA-3′; Reverse, 5′-AAGACTGTGTGCTCCCCATT-3′); β-actin (Forward, 5′-TCATGAAGTGTGACGTGGACATC-3′; Reverse, 5′-CAGGAGGAGCAATGATCTTGATCT-3′); miR-200b (Forward, 5′-GCGGCTAATACTGCCTGGTAA-3′; Reverse, 5′- GTGCAGGGTCCGAGGT-3′); miR-200c (Forward, 5′-TAATACTGCCGGGTAATGATGGA-3′; Reverse, 5′-CCAGTGCAGGGTCCGAGGT-3′); U6 (Forward, 5′-CTCGCTTCGGCAGCACA-3′; Reverse, 5′-AACGCTTCACGAATTTGCGT-3′). The relative expression levels of circDENND4C, miR-200b and miR-200c were determined with β-actin and U6 as internal control respectively by 2^-ΔΔCt^ method [18].

### Glucose consumption and lactate production

Transfected or non-transfected MDA-MB-453 and SK-BR-3 cells (1 × 10^5^/well) were seeded into 6-well plates overnight and then incubated in hypoxia or normoxia condition for 48 h. Glucose Assay Kit and Lactate Assay Kit (Sigma, St. Louis, MO, USA) were used for detection of glucose consumption and lactate production respectively following the manufacturer’s protocol. The relative levels of glucose consumption and lactate production in all treated groups were normalized to normoxia group.

### Western blot

After the indicated treatment, MDA-MB-453 and SK-BR-3 cells were collected and lysed in RIPA lysis buffer (Beyotime, Shanghai, China). The lysates were centrifuged at 12,000 g for 10 min and then total protein in supernatant was qualitied by using BCA protein assay kit (Beyotime). Following the boiled water bath, the equal amounts of proteins (30 μg) were loaded on SDS-PAGE gel and transferred to PVDF membranes (Millipore, Billerica, MA, USA). After blocking the non-specific binding sites with 5% non-fat milk, the membranes were probed with antibodies against hexokinase II (HK2) (ab227198, 1:5000, 102 kDa), matrix metallopeptidase 9 (MMP9) (ab38898, 1:1000, 92 kDa), MMP2 (ab97779, 1:1000, 72 kDa) or β-actin (ab8227, 1:3000, 42 kDa) overnight at 4 °C, along with horseradish peroxidase-labeled IgG (ab6721, 1:10000) for 2 h at room temperature. The antibodies used in this study were purchased from Abcam (Cambridge, MA, USA). The blots were visualized using BeyoECL Plus (Beyotime) and the relative protein level with β-actin as an endogenous control was normalized to normoxia group.

### Trans-well assay

For migration and invasion assays, trans-well assay was performed using 24-well trans-well chamber pre-coated with or without Matrigel (BD Bioscience, San Jose, CA, USA). Treated MDA-MB-453 and SK-BR-3 cells (4 × 10^4^/well) in serum-free RPMI-1640 medium were added to the upper chamber and 10% fetal bovine serum medium was added in the lower chamber. Cells were cultured at 37 °C with 5% CO_2_ for 24 h and then those on the lower surface were fixed with methanol (Sigma) and stained with 1% crystal violet (Sigma). A 200X magnification microscope (Olympus, Tokyo, Japan) was used to photograph the migrated and invasive cells with three randomly selected fields.

### Bioinformatics analysis, luciferase assay and RNA immunoprecipitation (RIP)

Bioinformatics analysis predicted the binding sites of circDENND4C and miR-200b or miR-200c by using starBase. The sequences of circDENND4C containing the predicted complementary sites of miR-200b or miR-200c (GCAGUAUU) at chr9: 1934268–1,934,275 were inserted into pmirGLO vectors (Promega, Madison, WI, USA) to generate wild-type luciferase reporter vector circDENND4C-WT (circ-WT). The corresponding mutant was generated by mutating the seed sites to AUGAGCAG, named as circDENND4C-MUT (circ-MUT). Luciferase assay was performed in MDA-MB-453 and SK-BR-3 cells co-transfected with 200 ng circ-WT or circ-MUT and 30 nM miR-200b, miR-200c or miR-NC using Lipofectamine 3000. A luciferase reporter assay kit (Promega) was used for the luciferase activity analysis at 24 h after the transfection.

For RIP assay, MDA-MB-453 and SK-BR-3 cells transfected with miR-200b, miR-200c or miR-NC were lysed in RIP lysis buffer. The combination of circDENND4C and miR-200b or miR-200c was examined by using the Magna RNA immunoprecipitation kit (Millipore) according to the manufacturer’s instructions. The magnetic beads were pre-coated by antibody against Ago2 (ab32381, Abcam) or IgG (AP112, Sigma). The level of circDENND4C (circ) enriched by RIP was measured by qRT-PCR.

### Murine xenograft model

The lentiviral vectors with sh-circDENND4C (sh-circ) or corresponding control (sh-NC) were constructed by FulenGen (Guangzhou, China). MDA-MB-453 cells were infected with sh-circ or sh-NC for 6 h and then stably transfected cells were selected under fluorescence microscope (Olympus) and flow cytometry (BD Bioscience). Five-week-old female BALB/c nude mice (Vital River Laboratory Animal Technology, Beijing, China) were injected subcutaneously with stably transfected MDA-MB-453 cells (5 × 10^6^), termed as sh-circ or sh-NC group (*n* = 3 per group). The mice injected with non-transfected cells were classified as empty group. Tumor volume was monitored every week and calculated using the formula: volume (mm^3^) = width^2^ × length/2. After 5 weeks following the inoculation, the mice were killed and tumor samples were weighted and harvested for measurement of circDENND4C, miR-200b and miR-200c expression levels. The experiment was permitted by the Animal Research Committee of the Third Affiliated Hospital of Zhengzhou University and performed in accordance with the guidelines of use of laboratory animals.

### Statistical analysis

GraphPad Prism 7 software (GraphPad Inc., La Jolla, CA, USA) was used for statistical analyses. Data from three independent experiments were expressed as mean ± standard deviation (S.D.). The comparison between two or more groups was performed by using paired student’s t test or one-way ANOVA with Tukey’s post hoc test. The association between circDENND4C level and clinicopathologic features of breast cancer patients was analyzed by χ^2^ test. *P < 0.05, **P < 0.01 and ***P < 0.001 were considered significant.

## Results

### The expression of circDENND4C is increased in breast cancer cells under hypoxia

**Fig. 1:**
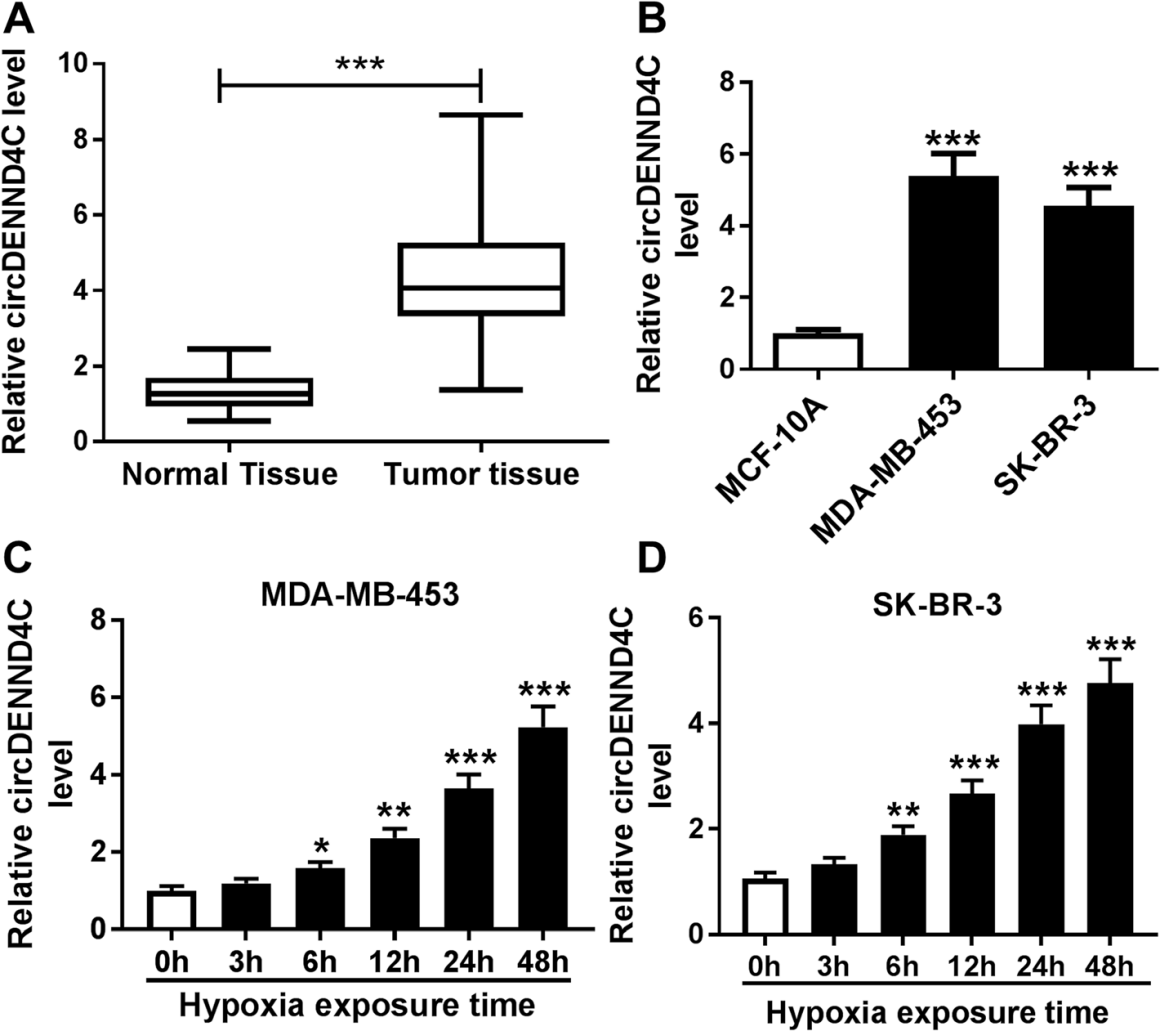
The expression of circDENND4C is enhanced in breast cancer under hypoxia. **a** circDENND4C expression in breast cancer tissues and adjacent normal samples was measured by qRT-PCR. *n* = 43. **b** The level of circDENND4C was detected in breast cancer cells (MDA-MB-453 and SK-BR-3) and normal cells (MCF-10A) by qRT-PCR. **c** and **d** The abundance of circDENND4C was examined in MDA-MB-453 and SK-BR-3 cells after hypoxia exposure for various time by qRT-PCR. *P < 0.05, **P < 0.01, ***P < 0.001

To explore the role of circDENND4C (the scheme of circDENND4C was shown in Additional file 1: Figure. S1) in breast cancer, its expression was measured in breast cancer tissues and cells. The pathologies of tumor tissues and normal samples were displayed in Additional file 2: Figure S2. As shown in Fig. 1a and Additional file 3: Figure S3A, compared with corresponding adjacent normal tissues, tumor tissues (*n* = 43) displayed significantly increased level of circDENND4C. More specifically, circDENND4C level was significantly increased in the four subtypes of breast cancer (TNBC, HER2, Luminal A and B) compared with that in normal tissues (Additional file 4: Figure S4). Moreover, by dividing into two groups according to the mean value of circDENND4C level, the high expression of circDENND4C was associated with tumor node metastasis stage, lymph node metastasis and tumor size (*P* < 0.05) but not with age, menopause and subtype (*P* > 0.05) (Table 1). In addition, the expression of circDENND4C was markedly enhanced in breast cancer cells (MDA-MB-453 and SK-BR-3) compared with that in MCF-10A cells (Fig. 1b and Additional file 5: Figure S5). Furthermore, the expression of circDENND4C was detected in MDA-MB-453 and SK-BR-3 cells after exposure of hypoxia. Results showed that HIF1A (a hypoxia indicator) protein level was significantly increased in cells after treatment of hypoxia (Additional file 6: Figure S6A and 6B) and the abundance of circDENND4C was progressively enhanced in MDA-MB-453 and SK-BR-3 cells after exposure of hypoxia in a time dependent manner (Fig. 1c and d). Besides, we confirmed the expression of circDENND4C was associated with HIF1A level in the two cell lines (Additional file 7: Figure S7A-7F).

### Knockdown of circDENND4C inhibits glycolysis, migration and invasion in breast cancer cells under hypoxia

**Fig. 2:**
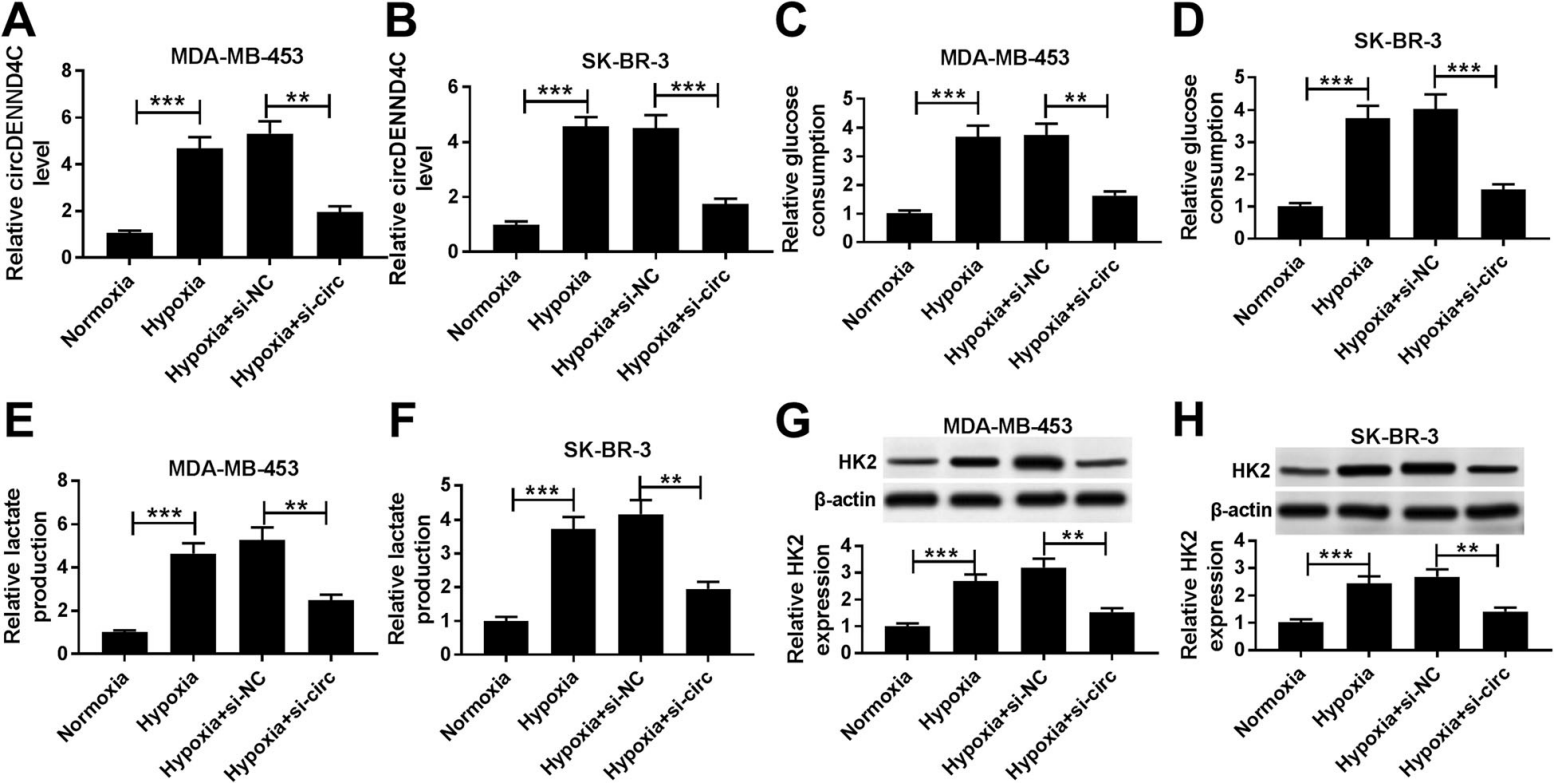
Silence of circDENND4C inhibits glycolysis in breast cancer cells under hypoxia. The expression level of circDENND4C (**a** and **b**), glucose consumption (**c** and **d**), lactate production (**e** and **f**) and HK2 protein level (**g** and **h**) were measured in MDA-MB-453 and SK-BR-3 cells transfected with siRNA against circDENND4C (si-circ) or siRNA negative control (si-NC) after treatment of hypoxia by qRT-PCR, special commercial kits and western blot, respectively. **P < 0.01, ***P < 0.001

**Fig. 3:**
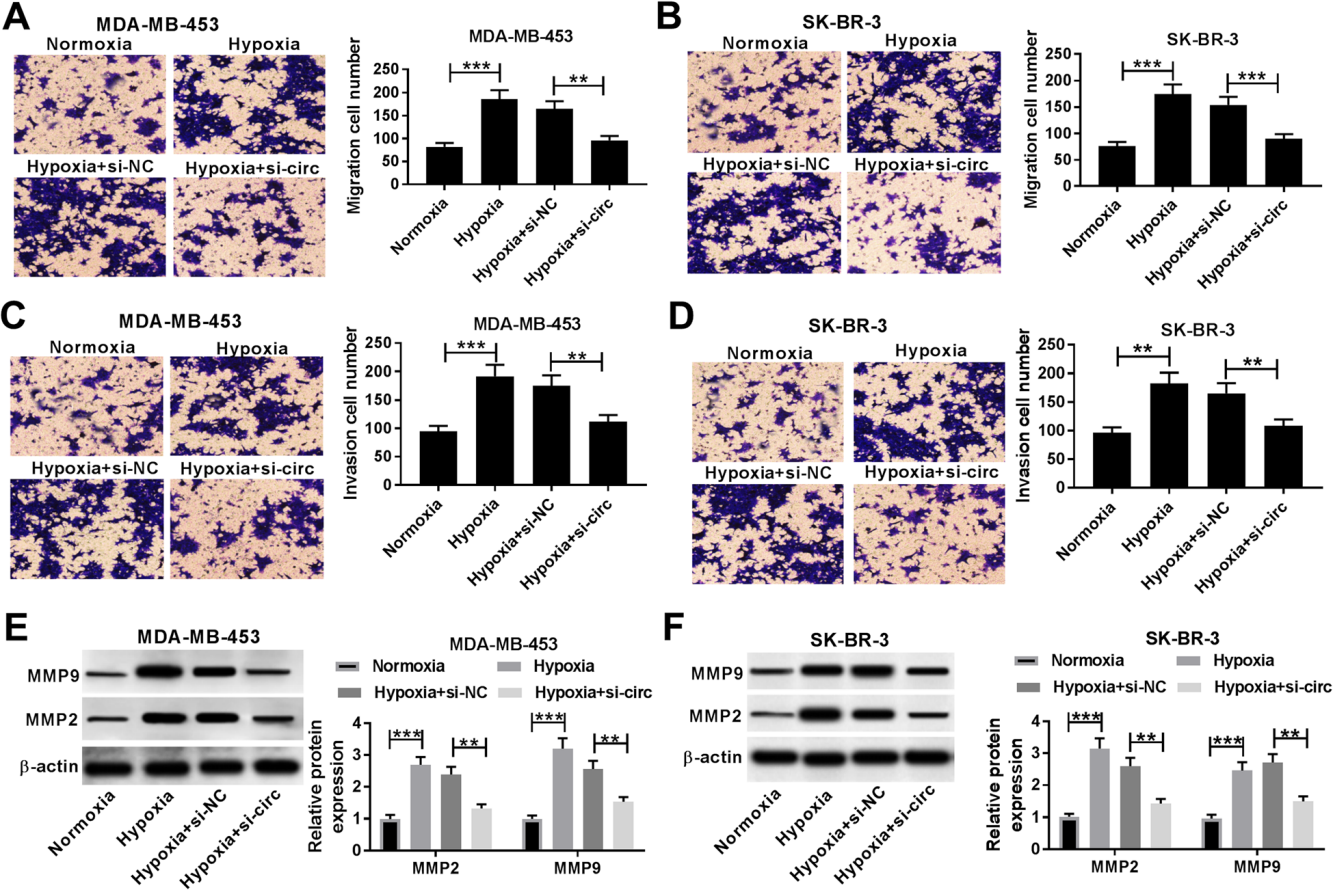
Knockdown of circDENND4C suppresses migration and invasion in breast cancer cells under hypoxia. Cell migration, invasion (**a**-**d**) and the protein levels of MMP9 and MMP2 (**e** and **f**) were detected in MDA-MB-453 and SK-BR-3 cells transfected with siRNA against circDENND4C (si-circ) or siRNA negative control (si-NC) after treatment of hypoxia by trans-well assay and western blot. **P < 0.01, ***P < 0.001

To investigate the effect of circDENND4C on breast cancer progression, its level was knocked down using si-circ in MDA-MB-453 and SK-BR-3 cells under hypoxia of 48 h (Fig. 2a and b). Moreover, treatment of hypoxia of 48 h induced obvious increase of glucose consumption, lactate production and HK2 protein level in MDA-MB-453 and SK-BR-3 cells, while silence of circDENND4C greatly weakened these events (Fig. 2c-h). Furthermore, the analyses of trans-well described that interference of circDENND4C notably decreased the abilities of cell migration and invasion promoted by hypoxia treatment in MDA-MB-453 and SK-BR-3 cells under hypoxia (Fig. 3a-d). However, overexpression of circDENND4C showed little effect on migration and invasion of these two cell lines under normoxia (Additional file 8: Figure S8A-8C). In addition, the protein expression levels of MMP9 and MMP2 in the two cells were conspicuously elevated by hypoxia exposure, which was counteracted by silence of circDENND4C (Fig. 3e and f).

### miR-200b and miR-200c are sponged by circDENND4C

**Fig. 4:**
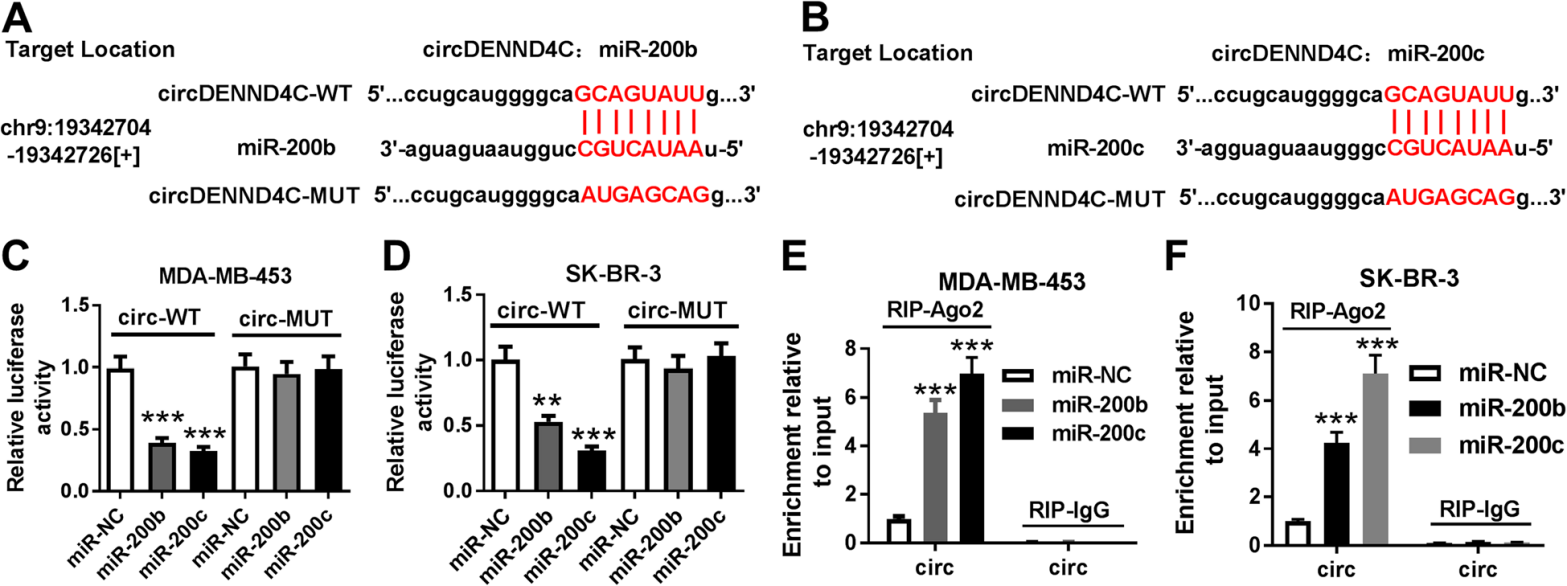
circDENND4C is a sponge of miR-200b/c. **a** and **b** The potential binding sites of circDENND4C and miR-200b/c were predicted by starBase. **c** and **d** Luciferase activity was analyzed in MDA-MB-453 and SK-BR-3 cells co-transfected with miR-200b mimic (miR-200b), miR-200c mimic (miR-200c) or miRNA negative control (miR-NC) and wild-type (WT) or mutant (MUT) circDENND4C luciferase reporter vector (circ-WT or circ-MUT). **e** and **f** The level of circDENND4C (circ) enriched by RIP was detected in MDA-MB-453 and SK-BR-3 cells transfected with miR-200b, miR-200c or miR-NC. **P < 0.01, ***P < 0.001

To elucidate the potential regulatory mechanism underlying circDENND4C in breast cancer progression, its target miRNAs were explored. As shown in Fig. 4a and b, bioinformatics analysis using starBase provided the predicted binding sites of circDENND4C and miR-200b or miR-200c. To validate this prediction, luciferase assay and RIP assay were performed in MDA-MB-453 and SK-BR-3 cells. As demonstrated in Fig. 4c and d, overexpression of miR-200b or miR-200c led to great loss of luciferase activity in circ-WT group, while they did not affect the activity in circ-MUT group. Furthermore, addition of miR-200b or miR-200c resulted in higher level of circDENND4C enriched by Ago2 RIP in MDA-MB-453 and SK-BR-3 cells than transfection of miR-NC, whereas IgG group showed little enrichment of circDENND4C (Fig. 4e and f).

### miR-200b and miR-200c levels are decreased in breast cancer under hypoxia and negatively regulated by circDENND4C

**Fig. 5:**
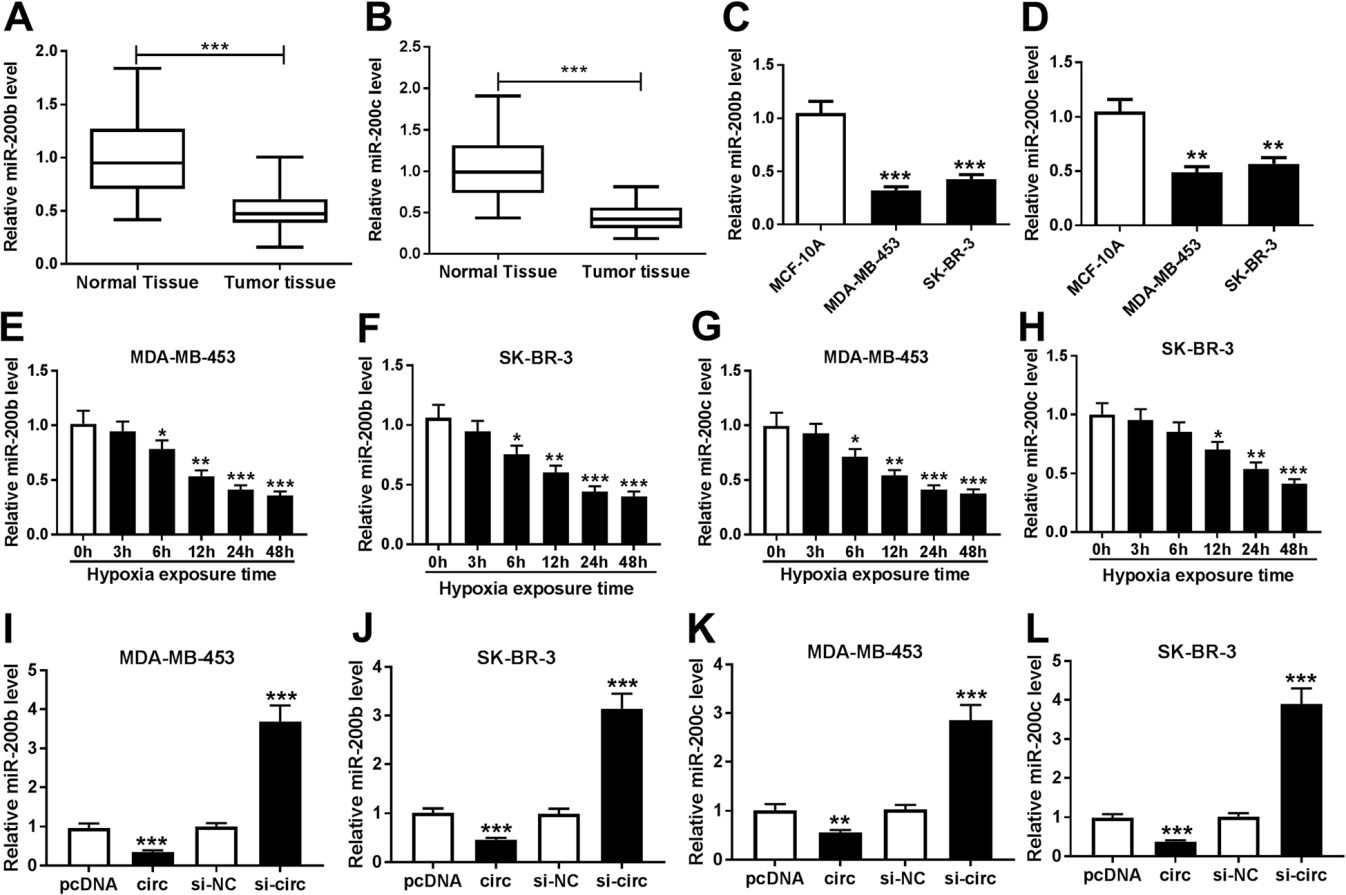
miR-200b/c levels are down-regulated in breast cancer under hypoxia and negatively regulated by circDENND4C. **a**-**d** miR-200b/c expression levels in breast cancer tissues (*n* = 43) and cells were measured by qRT-PCR. **e**-**h** The abundances of miR-200b and miR-200c were detected in MDA-MB-453 and SK-BR-3 cells after exposure of hypoxia. **i**-**l** The expression levels of miR-200b and miR-200c were measured in MDA-MB-453 and SK-BR-3 cells transfected with pcDNA, circDENND4C overexpression vector (circ), siRNA negative control (si-NC) or siRNA against circDENND4C (si-circ). *P < 0.05, **P < 0.01, ***P < 0.001

To explore the role of miR-200b and miR-200c in breast cancer, their expression levels were detected. As shown in Fig. 5a and b and Additional file 3: Figure S3B and 3C, the expression levels of miR-200b and miR-200c were evidently reduced in breast cancer tissues (*n* = 43) compared with those in normal samples. Moreover, their levels were also decreased in MDA-MB-453 and SK-BR-3 cells in comparison to those in MCF-10A cells (Fig. 5c and d). In addition, after stimulation of hypoxia condition, the abundances of miR-200b and miR-200c were progressively down-regulated in the two cells in a time dependent manner (Fig. 5e-h). Besides, qRT-PCR assay also revealed that the expression levels of miR-200b and miR-200c in MDA-MB-453 and SK-BR-3 cells were significantly decreased by overexpression of circDENND4C but increased via knockdown of circDENND4C (Fig. 5i-l).

### Knockdown of miR-200b or miR-200c reverses silence of circDENND4C-induced progression inhibition in breast cancer cells under hypoxia

**Fig. 6:**
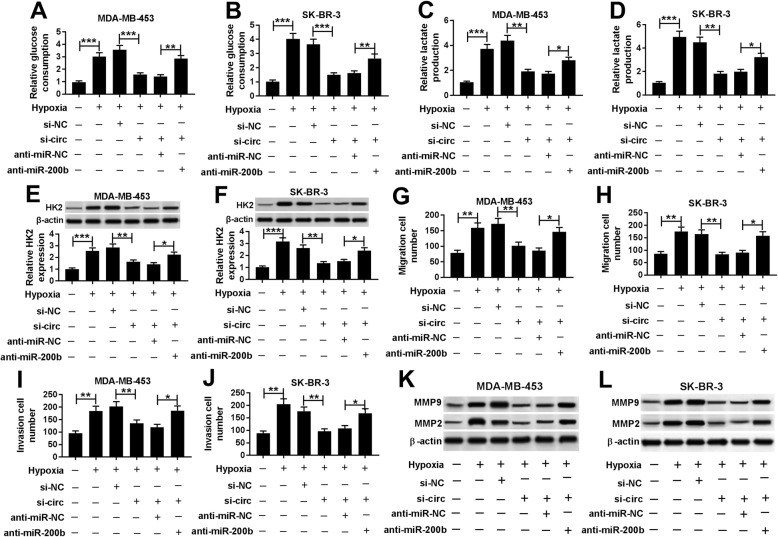
miR-200b deficiency reverses silence of circDENND4C-mediated progression inhibition in breast cancer cells under hypoxia. Glucose consumption (**a** and **b**), lactate production (**c** and **d**), HK2 protein level (**e** and **f**), cell migration (**g** and **h**), invasion (**i** and **j**) and protein levels of MMP9 and MMP2 (**k** and **l**) were measured in MDA-MB-453 and SK-BR-3 cells transfected with siRNA negative control (si-NC), siRNA against circDENND4C (si-circ), si-circ and miR-200b inhibitor (anti-miR-200b) or inhibitor negative control (anti-miR-NC) by special commercial kits, western blot and trans-well assay, respectively. *P < 0.05, **P < 0.01, ***P < 0.001

**Fig. 7:**
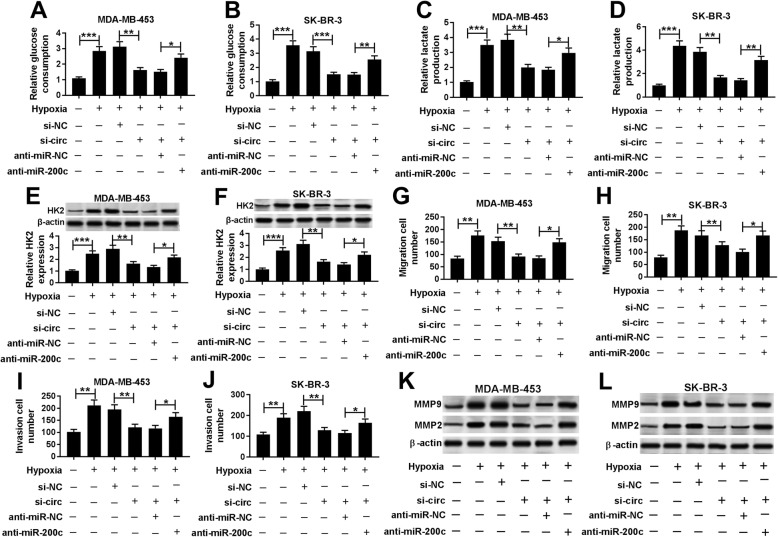
miR-200c exhaustion attenuates silence of circDENND4C-modulated progression suppression in breast cancer cells under hypoxia. Glucose consumption (**a** and **b**), lactate production (**c** and **d**), HK2 protein level (**e** and **f**), cell migration (**g** and **h**), invasion (**i** and **j**) and protein levels of MMP9 and MMP2 (**k** and **l**) were detected in MDA-MB-453 and SK-BR-3 cells transfected with siRNA negative control (si-NC), siRNA against circDENND4C (si-circ), si-circ and miR-200c inhibitor (anti-miR-200c) or inhibitor negative control (anti-miR-NC) by special commercial kits, western blot and trans-well assay, respectively. *P < 0.05, **P < 0.01, ***P < 0.001

To explore whether miR-200b was responsible for circDENND4C-mediated breast cancer progression, MDA-MB-453 and SK-BR-3 cells were transfected with si-NC, si-circ, si-circ and anti-miR-200b or anti-miR-NC before treatment of hypoxia. As shown in Fig. 6a-f, knockdown of miR-200b attenuated silence of circDENND4C-mediated inhibition of glucose consumption, lactate production and HK2 protein level in MDA-MB-453 and SK-BR-3 cells under hypoxia. Moreover, miR-200b deficiency alleviated the suppressive effect of circDENND4C knockdown on migration and invasion in the two cells under hypoxia (Fig. 6g-j). Additionally, the protein levels of MMP9 and MMP2 inhibited by circDENND4C interference were restored by miR-200b absence (Fig. 6k and l). Similarly, to explore whether circDENND4C-mediated breast cancer progression was modulated by miR-200c, MDA-MB-453 and SK-BR-3 cells were transfected with si-NC, si-circ, si-circ and anti-miR-200c or anti-miR-NC and then treated by hypoxia. Results showed that knockdown of miR-200c abated the inhibitive effect of circDENND4C silence on glucose consumption, lactate production, HK2 protein level, migration, invasion, MMP9 and MMP2 protein levels in MDA-MB-453 and SK-BR-3 cells under hypoxia (Fig. 7a-l).

### Knockdown of circDENND4C decreases xenograft tumor growth by increasing miR-200b and miR-200c

**Fig. 8:**
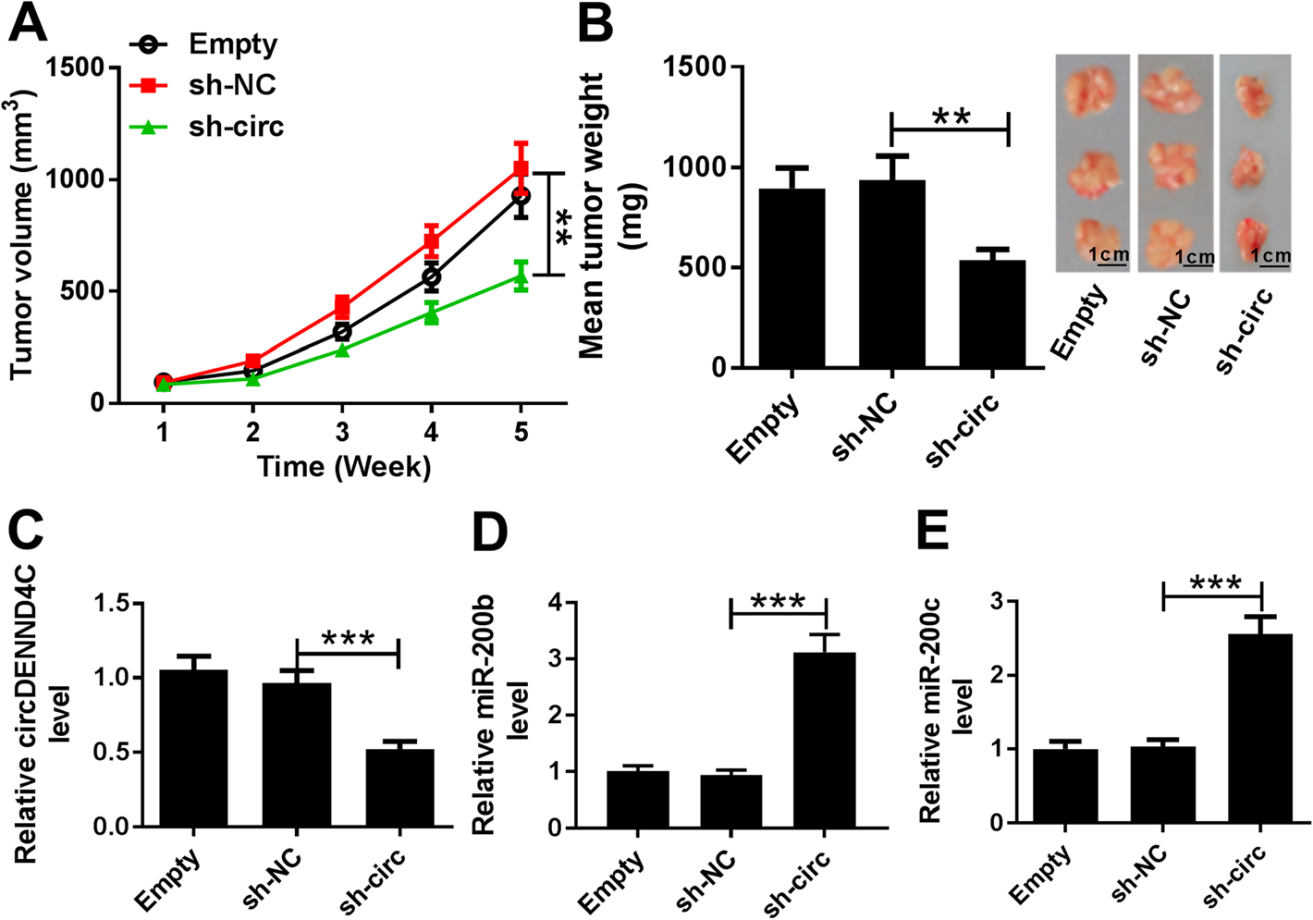
Knockdown of circDENND4C decreases breast cancer cell xenograft tumor growth. **a** and **b** Tumor volume and weight were measured in shRNA against circDENND4C (sh-circ), shRNA negative control (sh-NC) or non-transfected (Empty) group. **c**-**e** The expression levels of circDENND4C, miR-200b and miR-200c were detected in tumor tissues by qRT-PCR. **P < 0.01, ***P < 0.001

To further investigate the anti-cancer role of circDENND4C silence, MDA-MB-453 cells stably transfected with sh-circ or sh-NC or non-transfected cells (Empty) were used to establish xenograft model in vivo. The infection efficacy was confirmed in Additional file 9: Figure S9A and 9B. After cell injection for 5 weeks, tumor volume and weight were significantly reduced in sh-circ group compared with those in sh-NC or empty group (Fig. 8a and b). Meanwhile, HIF1A expression was notably decreased in sh-circ group compared with those in sh-NC or empty group (Additional file 10: Figure S10). Moreover, the expression of circDENND4C was decreased 48% in sh-circ group when compared with that in sh-NC group (Fig. 8c). However, the levels of miR-200b and miR-200c were increased 3.12-fold and 2.56-fold respectively in sh-circ group in comparison to those in sh-NC group (Fig. 8d and e).

## Discussion

Hypoxia is a key feature of cancers and circRNAs are involved in regulation of hypoxia [9]. Among all breast cancer-associated with circRNA, circDENND4C is a circRNA in response to hypoxia. This study was the first to investigate the effect of circDENND4C on glycolysis, migration and invasion in breast cancer and explore the potential sponging miRNAs.

To resist hypoxia stress, cancer cell would trigger hypoxia-inducible factor 1 alpha (HIF1A) expression, which is related with dysregulation of circRNA. Previous study suggested that circDENND4C is a HIF1A-associated circRNA, which is highly expressed in hypoxia condition and promotes cell proliferation at 4 days in breast cancer [10]. Similarly, we also found that circDENND4C expression was enhanced in breast cancer cells after treatment of hypoxia. Moreover, high expression of circDENND4C predicted poor outcomes of patients with breast cancer. The hypoxia environment would lead to the reprogramming of glycolytic metabolism, which is characterized by glucose consumption and lactate production. Furthermore, HK2 is a key enzyme associated with glycolysis in cancers, including breast cancer [19–21]. Under hypoxia, glucose consumption, lactate production and HK2 protein level were enhanced in breast cancer cells, indicating that glycolysis was triggered. However, loss-of-function experiments uncovered that circDENND4C knockdown could decrease glycolysis in breast cancer cells under hypoxia. Besides, hypoxia-induced epithelial mesenchymal transition is an important mechanism of tumor progression, which is responsible for cancer cell migration and invasion [22, 23]. MMPs, especially MMP9 and MMP2, are crucial biomarkers for migration and invasion in breast cancer [24–26]. By detecting MMPs levels and trans-well assay, we found that cell migration and invasion were increased in cells under hypoxia by increasing MMP2 and MMP9, which was inhibited by silence of circDENND4C. We hypothesized that anti-metastatic role of circDENND4C silence might be associated with epithelial mesenchymal transition, which needs further study in future. These indicated circDENND4C as a therapeutic target for breast cancer patients.

Prior studies have revealed that circRNAs could serve as sponges of miRNAs [27–29]. To figure out whether circDENND4C regulates breast cancer progression by sponging miRNAs, its targets were explored by bioinformatics analysis using starBase, predicting that miR-200b and miR-200c might be sponged by circDENND4C, which was confirmed by luciferase activity and RIP assays. Our research showed that miR-200b and miR-200c expression levels were decreased in breast cancer cells after exposure of hypoxia, uncovering that they might be associated with regulation of hypoxia. Previous studies demonstrated that miR-200b and miR-200c could inhibit glycolysis in cancers by targeting lactate dehydrogenase A and sirtuin 2 [30, 31]. Additionally, miR-200b and miR-200c could suppress migration and invasion of breast cancer cells by regulating ezrin-radixin-moesin and fucosyltransferase-4 [32, 33]. These reports suggested the anti-cancer roles of miR-200b and miR-200c by decreasing glycolysis, migration and invasion in human cancers. The current study using rescue experiments revealed that knockdown of miR-200b and miR-200c attenuated the anti-cancer role of circDENND4C silence in breast cancer under hypoxia, indicating that circDENND4C regulates breast cancer progression by sponging miR-200b and miR-200c. Besides, in vivo experiments further supported the suppressive effect of circDENND4C interference by increasing miR-200b and miR-200c. The function of miRNAs is realized by regulating mRNA expression, hence the promising targets of miR-200b and miR-200c should be explored in further study. Besides, hypoxia is regulated not only by HIF1A, but also via mTORC1 signaling. Here we found that knockdown of circDENND4C suppressed the activation of p70S6K1, a marker of mTORC1 signaling (Additional file 11: Figure S11A and 11B), indicating the importance of this pathway for mechanism mediated by circDENND4C. While the more details need further study in future.

## Conclusion

In summary, this study highlighted circDENND4C as a novel prognostic target for breast cancer and elucidated that knockdown of circDENND4C plays an anti-cancer role through suppressing glycolysis, migration and invasion in breast cancer under hypoxia, possibly by increasing miR-200b and miR-200c. This work provides a new mechanism for understanding cancer progression under hypoxia and indicates circDENND4C as a promising therapeutic target for breast cancer.

## Additional files

Additional file 1: **Figure S1.** The scheme of circDENND4C. (TIF 392 kb)

Additional file 2: **Figure S2.** The pathology of breast cancer tissues was analyzed by hematoxylin and eosin (HE) staining. (TIF 853 kb)

Additional file 3: **Figure S3.** The levels of circDENND4C and miR-200b/c in breast cancer. (A-C) The expressions of circDENND4C (A), miR-200b (B) and miR-200c (C) were detected in tumor tissues and normal tissues by chromogenic in-situ hybridization assay. (TIF 3091 kb)

Additional file 4: **Figure S4.** The expression of circDENND4C in different types of breast cancer. ***P < 0.001. (TIF 111 kb)

Additional file 5: **Figure S5.** The expression of circDENND4C in breast cancer cells by chromogenic in-situ hybridization assay. (TIF 2269 kb)

Additional file 6: **Figure S6.** The expression of HIF1A in breast cancer cells after treatment of hypoxia. (A and B) The protein level of HIF1A was detected in MDA-MB-453 and SK-BR-3 cells after treatment of hypoxia for 0, 3, 6, 12, 24 and 48 h. *P < 0.05, **P < 0.01, ***P < 0.001. (TIF 276 kb)

Additional file 7: **Figure S7.** The effect of HIF1A on circDENND4C level. (A and B) The protein level of HIF1A was measured in MDA-MB-453 and SK-BR-3 cells with transfection of si-NC or si-circ and treatment of hypoxia. circDENND4C expression was detected in MDA-MB-453 and SK-BR-3 cells transfected with si-HIF1A under normoxia (C and D) or si-HIF1A under hypoxia (E and F). *P < 0.05, **P < 0.01, ***P < 0.001. (TIF 727 kb)

Additional file 8: **Figure S8.** The effect of circDENND4C on migration and invasion of breast cancer cells under normoxia. circDEND4C expression (A), migration (B) and invasion (C) were detected in MDA-MB-453 and SK-BR-3 cells transfected with pcDNA or circDENND4C under normoxia. **P < 0.01, ***P < 0.001, NS: not significant. (TIF 208 kb)

Additional file 9: **Figure S9.** The validation of stable silencing system in MDA-MB-453 cells. MDA-MB-453 cells were transfected with sh-NC, sh-circ or empty, and then the expression of circDENND4C (A) and infection efficiency (B) were analyzed by qRT-PCR or fluorescence microscope. ***P < 0.001. (TIF 961 kb)

Additional file 10: **Figure S10.** The expression of HIF1A in xenograft model by immunohistochemistry. (TIF 854 kb)

Additional file 11: **Figure S11.** The effect of circDENND4C on mTORC1 signaling. (A and B) The protein levels of p-p70S6K1 and p70S6K1 were detected in MDA-MB-453 and SK-BR-3 cells transfected with si-NC or si-circ after treatment of hypoxia. (TIF 301 kb)

## Data Availability

Please contact author for data request.

## Abbreviations

circDENND4C: circRNA differentially expressed in normal cells and neoplasia domain containing 4C
MMP9: Matrix metallopeptidase 9
qRT-PCR: Quantitative real-time polymerase chain reaction
RIP: RNA Immunoprecipitation
si-NC: siRNA negative control
siRNA: Small interfering RNA
SOCS3: Suppressor of cytokine signaling 3
