# Supplementary figures and images for: Knockdown of circDENND4C inhibits glycolysis, migration and invasion by up-regulating miR-200b/c in breast cancer under hypoxia
Source: J Exp Clin Cancer Res. 2019 Sep 5;38:388. doi: 10.1186/s13046-019-1398-2 (PMC6727545; doi:10.1186/s13046-019-1398-2)

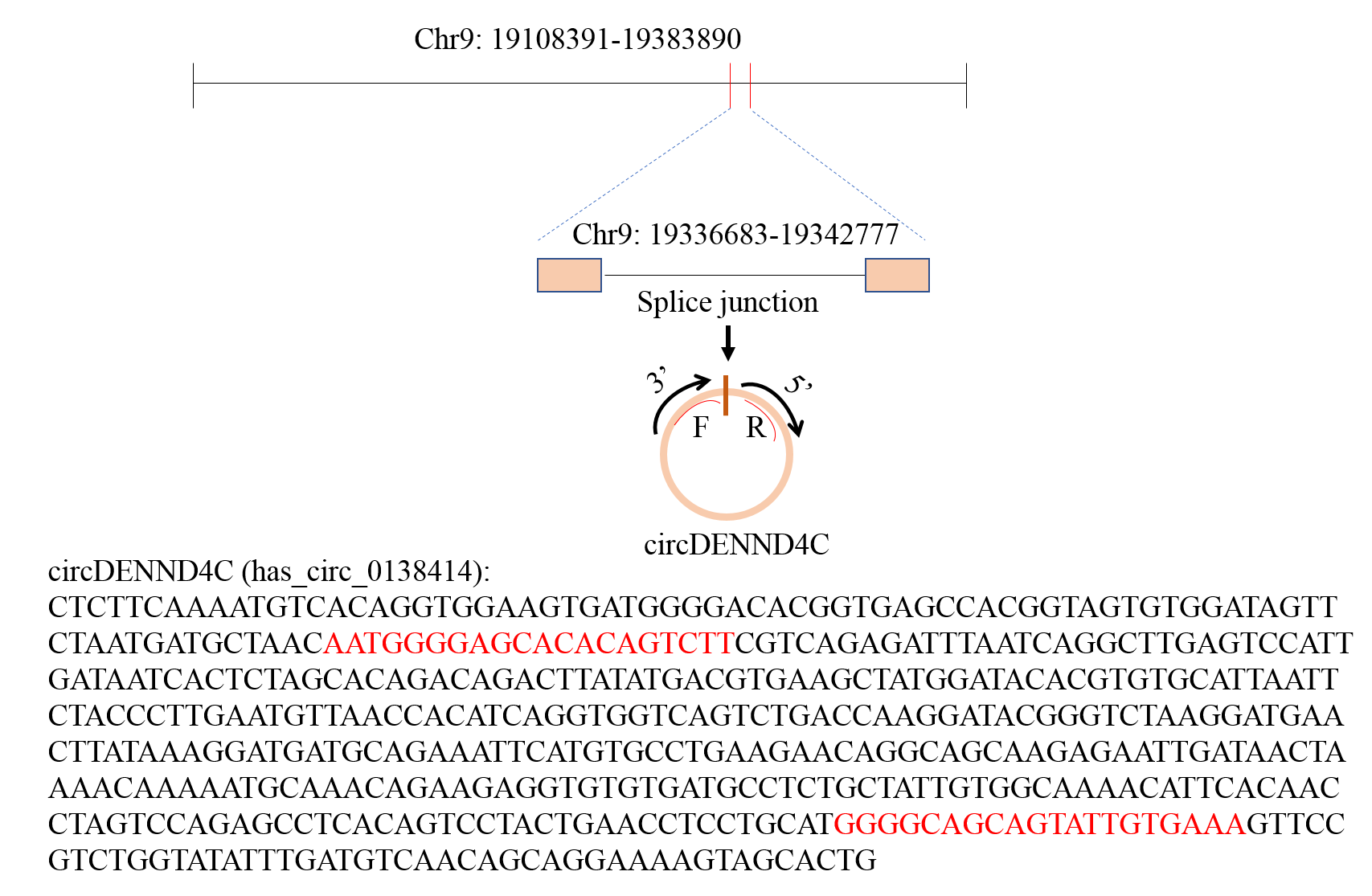

Supplement: Supplementary file 1 — Figure S1. The scheme of circDENND4C. (TIF 392 kb) [file 13046_2019_1398_MOESM1_ESM.tif]

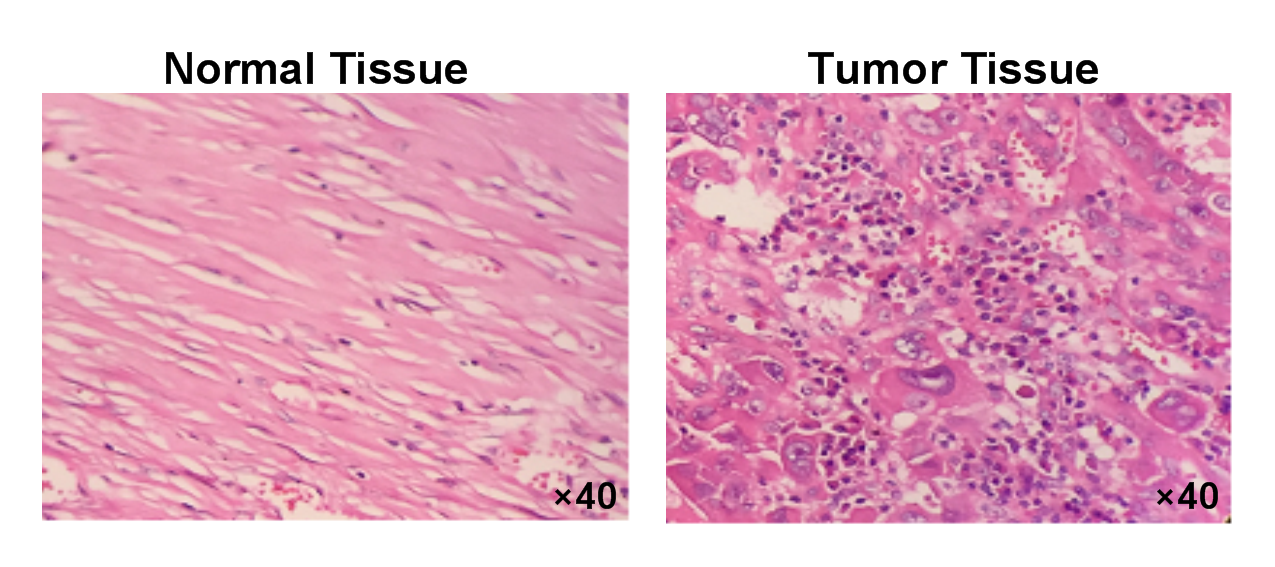

Supplement: Supplementary file 2 — Figure S2. The pathology of breast cancer tissues was analyzed by hematoxylin and eosin (HE) staining. (TIF 853 kb) [file 13046_2019_1398_MOESM2_ESM.tif]

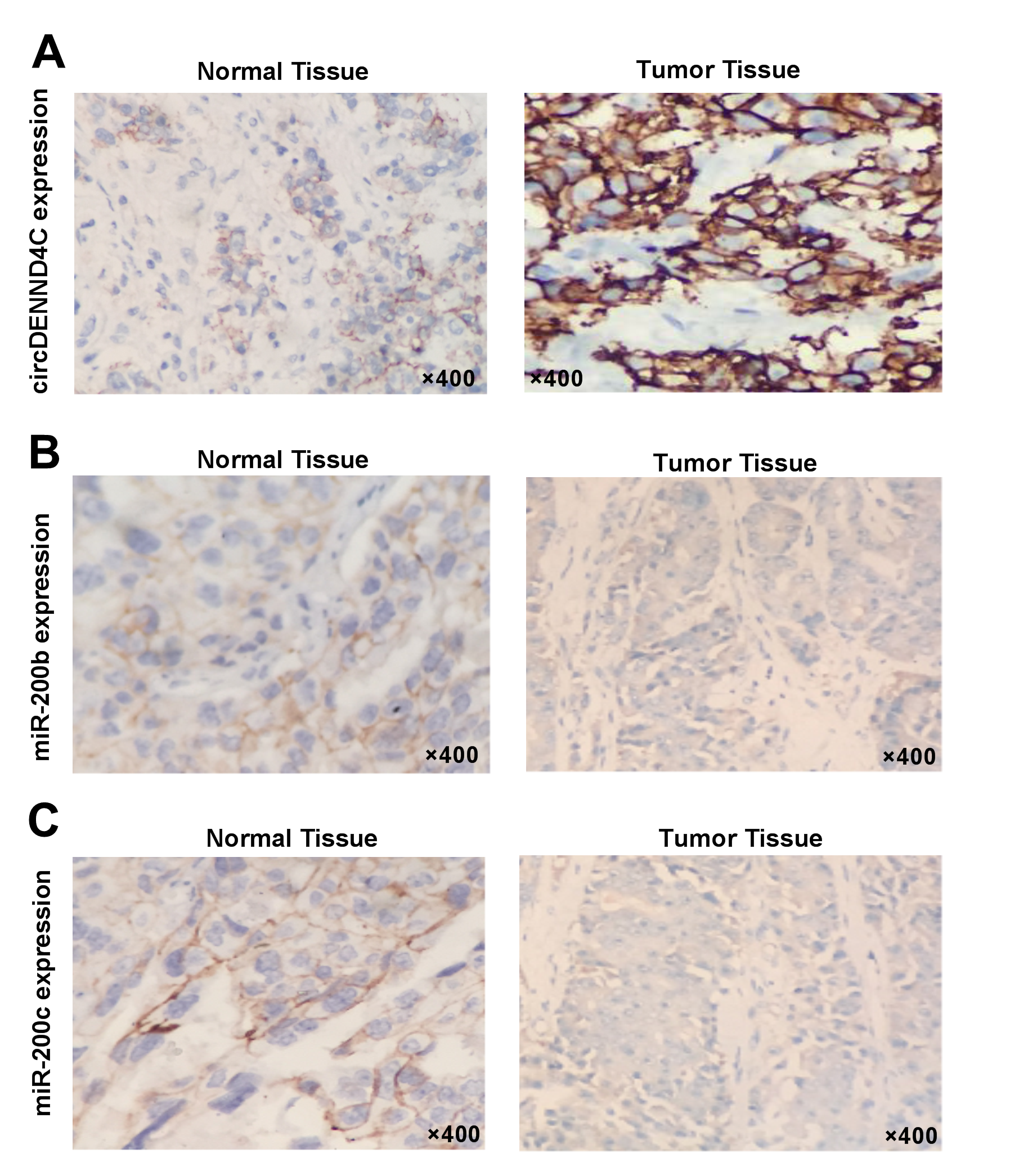

Supplement: Supplementary file 3 — Figure S3. The levels of circDENND4C and miR-200b/c in breast cancer. (A-C) The expressions of circDENND4C (A), miR-200b (B) and miR-200c (C) were detected in tumor tissues and normal tissues by chromogenic in-situ hybridization assay. (TIF 3091 kb) [file 13046_2019_1398_MOESM3_ESM.tif]

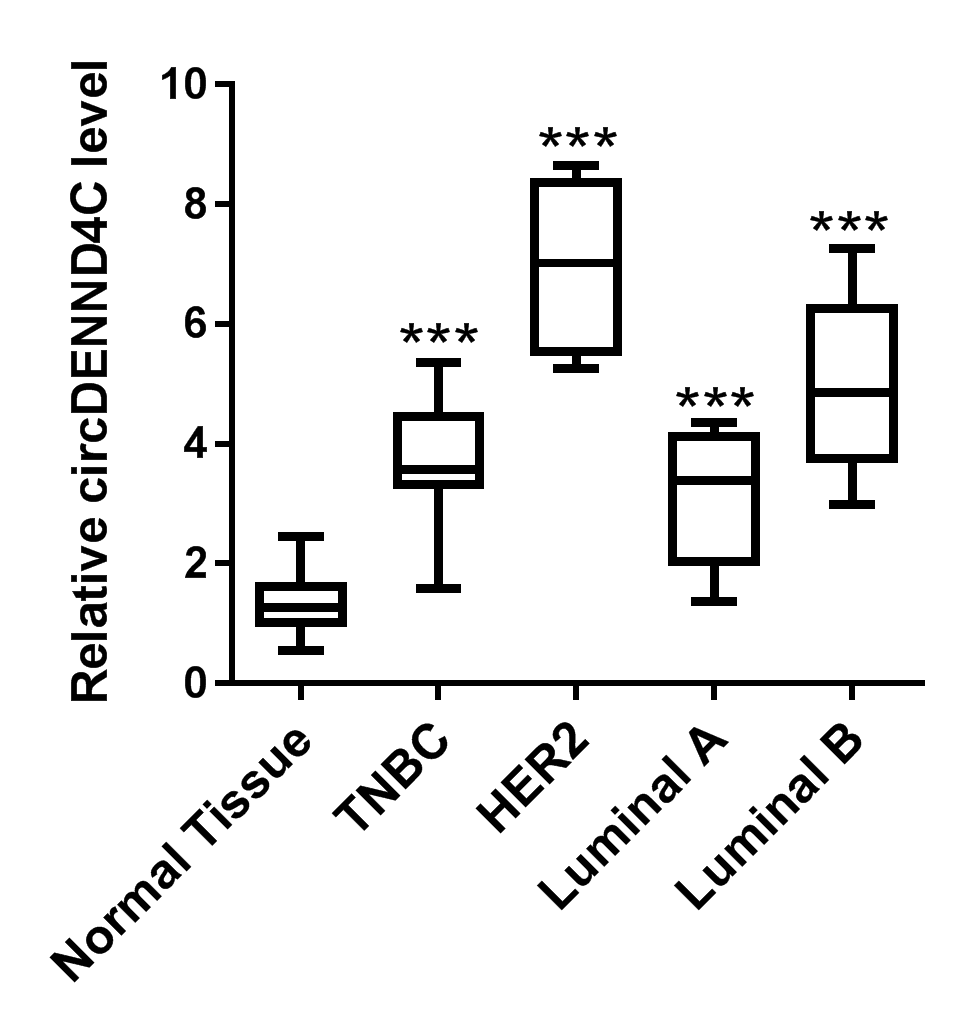

Supplement: Supplementary file 4 — Figure S4. The expression of circDENND4C in different types of breast cancer. ***P < 0.001. (TIF 111 kb) [file 13046_2019_1398_MOESM4_ESM.tif]

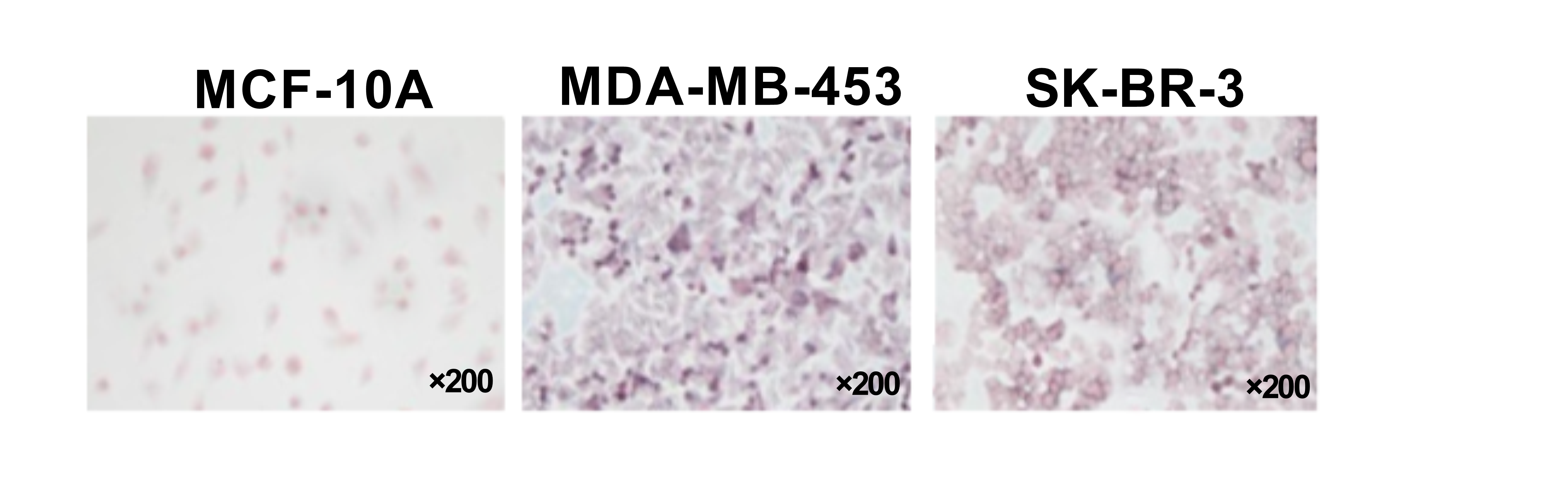

Supplement: Supplementary file 5 — Figure S5. The expression of circDENND4C in breast cancer cells by chromogenic in-situ hybridization assay. (TIF 2269 kb) [file 13046_2019_1398_MOESM5_ESM.tif]

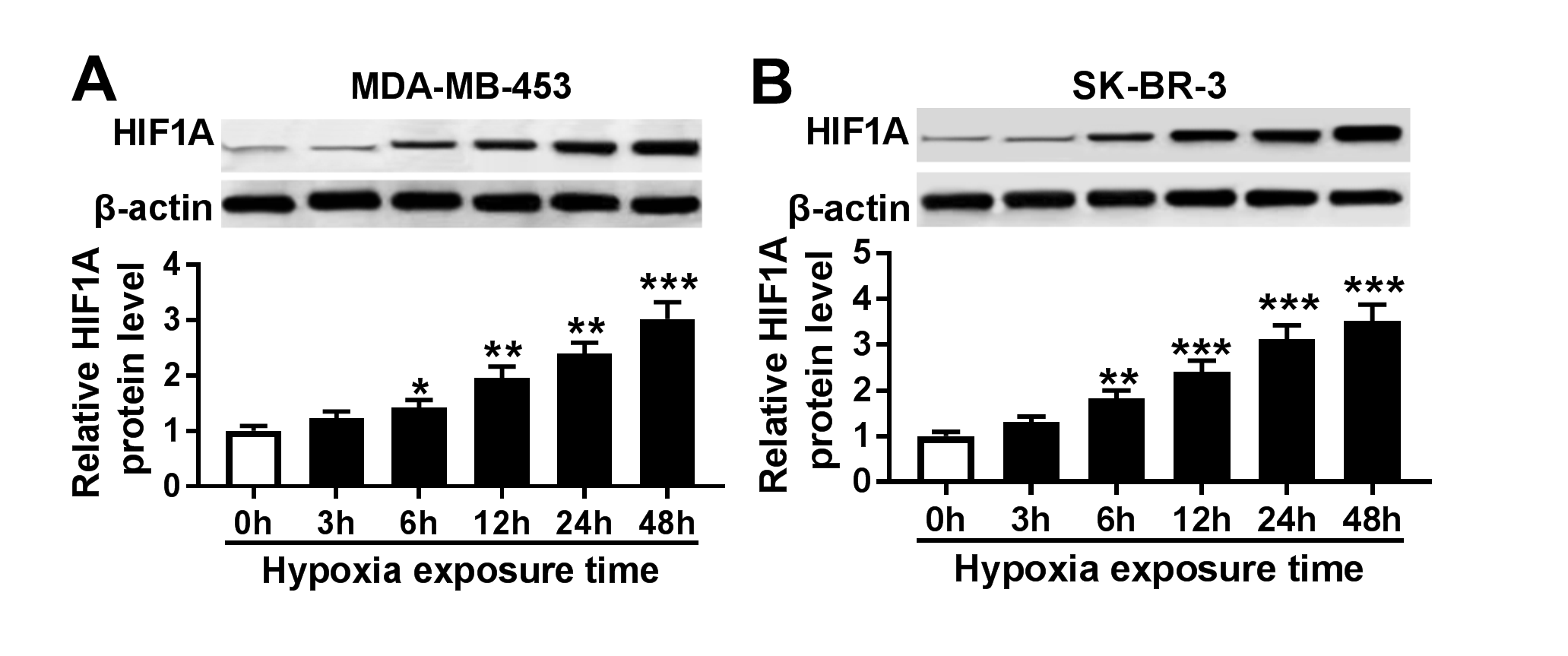

Supplement: Supplementary file 6 — Figure S6. The expression of HIF1A in breast cancer cells after treatment of hypoxia. (A and B) The protein level of HIF1A was detected in MDA-MB-453 and SK-BR-3 cells after treatment of hypoxia for 0, 3, 6, 12, 24 and 48 h. *P < 0.05, **P < 0.01, ***P < 0.001. (TIF 276 kb) [file 13046_2019_1398_MOESM6_ESM.tif]

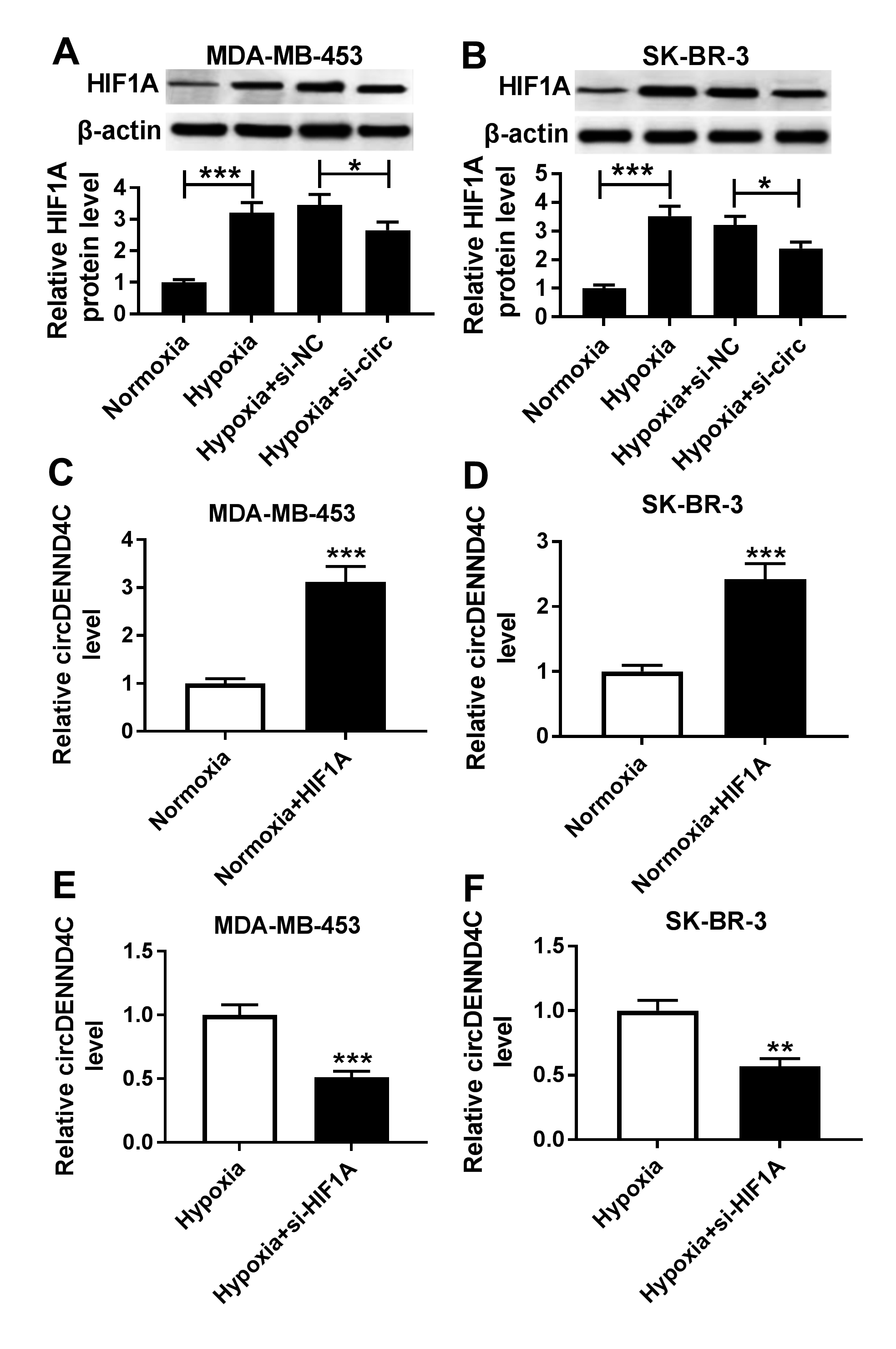

Supplement: Supplementary file 7 — Figure S7. The effect of HIF1A on circDENND4C level. (A and B) The protein level of HIF1A was measured in MDA-MB-453 and SK-BR-3 cells with transfection of si-NC or si-circ and treatment of hypoxia. circDENND4C expression was detected in MDA-MB-453 and SK-BR-3 cells transfected with si-HIF1A under normoxia (C and D) or si-HIF1A under hypoxia (E and F). *P < 0.05, **P < 0.01, ***P < 0.001. (TIF 727 kb) [file 13046_2019_1398_MOESM7_ESM.tif]

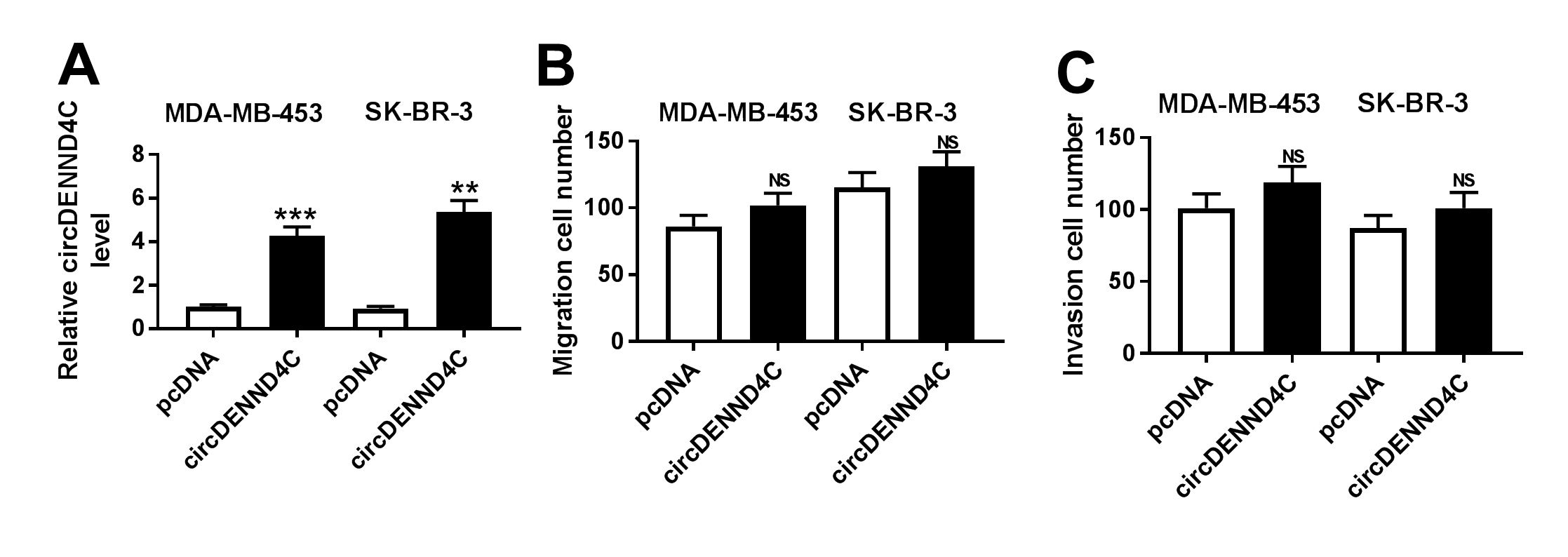

Supplement: Supplementary file 8 — Figure S8. The effect of circDENND4C on migration and invasion of breast cancer cells under normoxia. circDEND4C expression (A), migration (B) and invasion (C) were detected in MDA-MB-453 and SK-BR-3 cells transfected with pcDNA or circDENND4C under normoxia. **P < 0.01, ***P < 0.001, NS: not significant. (TIF 208 kb) [file 13046_2019_1398_MOESM8_ESM.tif]

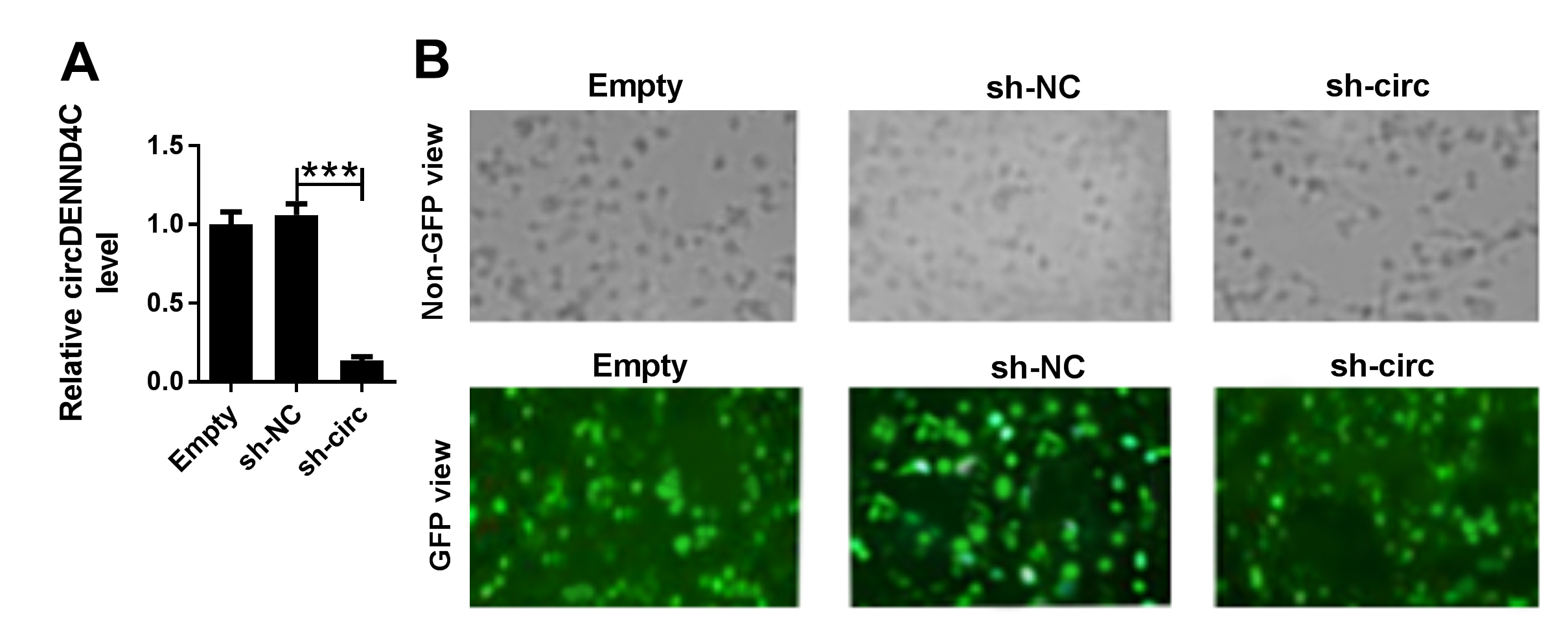

Supplement: Supplementary file 9 — Figure S9. The validation of stable silencing system in MDA-MB-453 cells. MDA-MB-453 cells were transfected with sh-NC, sh-circ or empty, and then the expression of circDENND4C (A) and infection efficiency (B) were analyzed by qRT-PCR or fluorescence microscope. ***P < 0.001. (TIF 961 kb) [file 13046_2019_1398_MOESM9_ESM.tif]

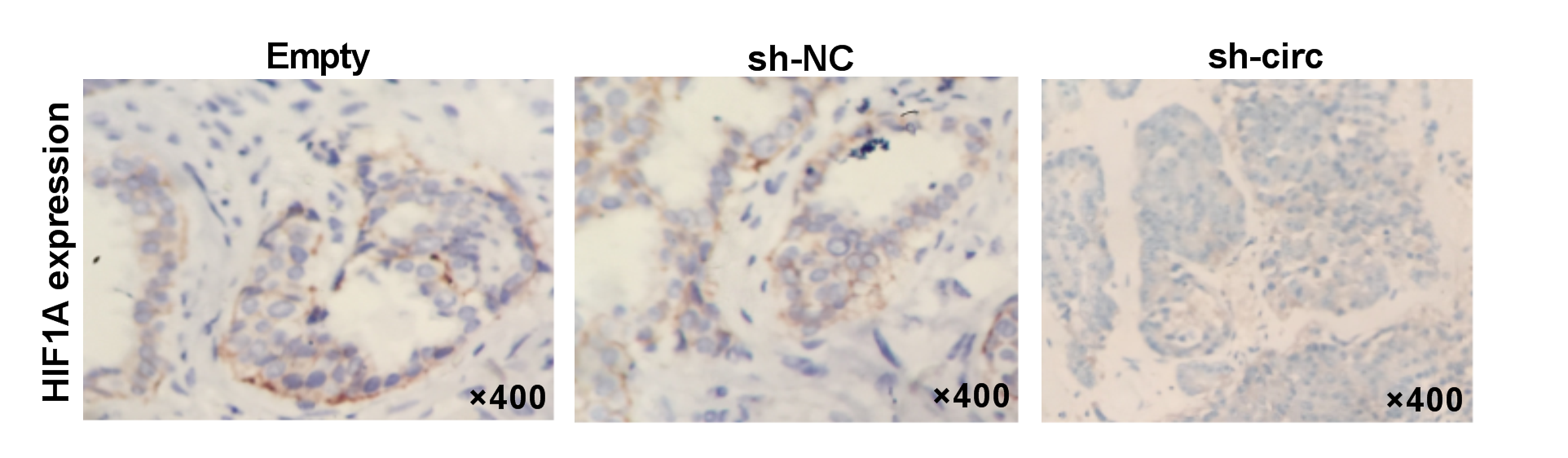

Supplement: Supplementary file 10 — Figure S10. The expression of HIF1A in xenograft model by immunohistochemistry. (TIF 854 kb) [file 13046_2019_1398_MOESM10_ESM.tif]

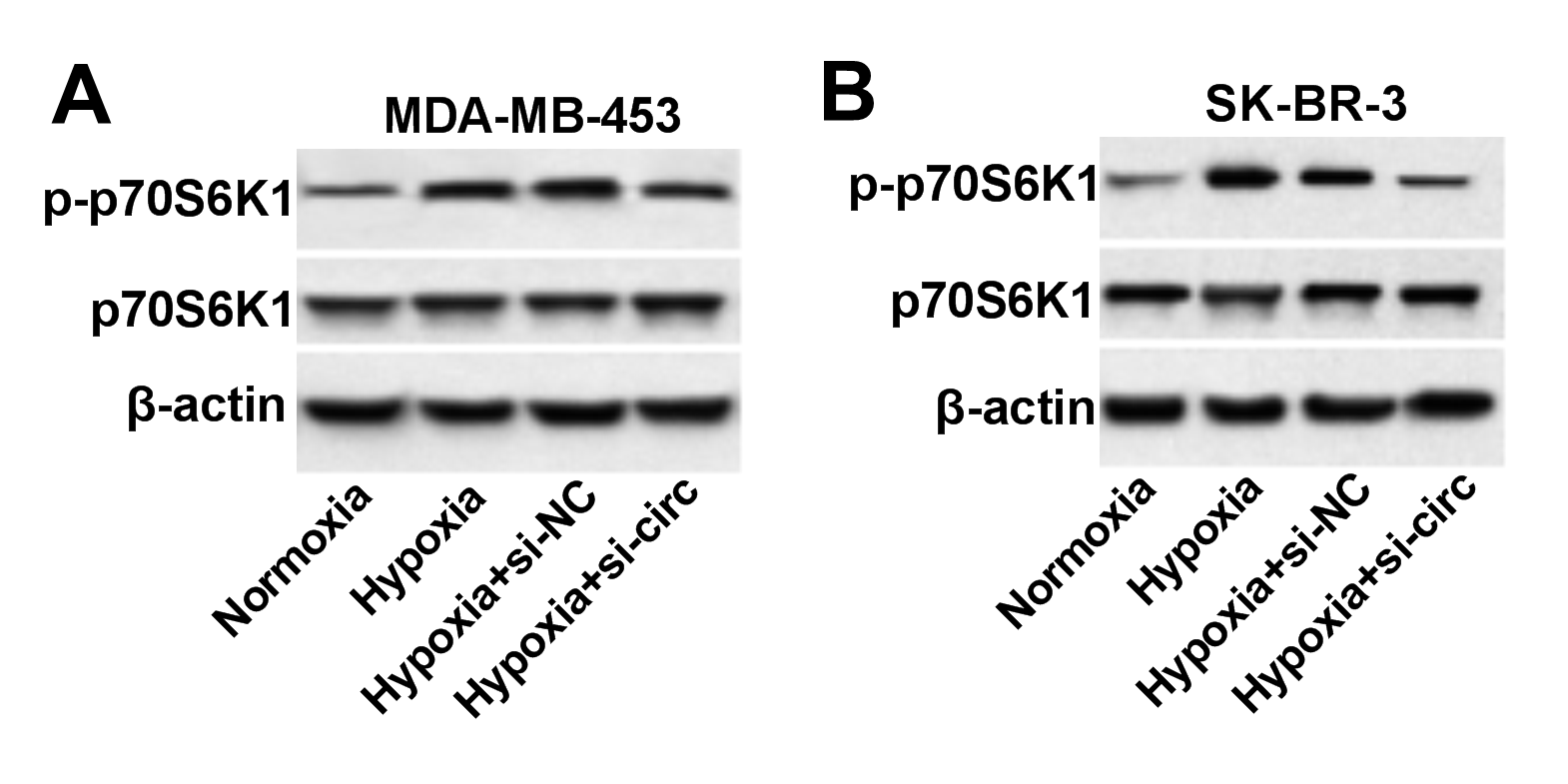

Supplement: Supplementary file 11 — Figure S11. The effect of circDENND4C on mTORC1 signaling. (A and B) The protein levels of p-p70S6K1 and p70S6K1 were detected in MDA-MB-453 and SK-BR-3 cells transfected with si-NC or si-circ after treatment of hypoxia. (TIF 301 kb) [file 13046_2019_1398_MOESM11_ESM.tif]
